# Lead I R‐wave amplitude to distinguish ventricular arrhythmias with lead V_3_ transition originating from the left versus right ventricular outflow tract

**DOI:** 10.1002/clc.23511

**Published:** 2020-12-10

**Authors:** Jue Wang, Chenglong Miao, Guangmin Yang, Lu Xu, Ru Xing, Yan Jia, Ruining Zhang, Yanwei Wang, Liu Huang, Suyun Liu

**Affiliations:** ^1^ Department of Cardiology Second Hospital of Hebei Medical University Shijiazhuang China; ^2^ Department of Joint Surgery Third Hospital of Hebei Medical University Shijiazhuang China

**Keywords:** catheter ablation, electrocardiography, lead I, outflow tract, ventricular arrhythmias

## Abstract

**Background:**

The electrophysiology algorithm for localizing left or right origins of outflow tract ventricular arrhythmias (OT‐VAs) with lead V_3_ transition still needs further investigation in clinical practice.

**Hypothesis:**

Lead I R‐wave amplitude is effective in distinguishing the left or right origin of OT‐VAs with lead V_3_ transition.

**Methods:**

We measured lead I R‐wave amplitude in 82 OT‐VA patients with lead V_3_ transition and a positive complex in lead I who underwent successful catheter ablation from the right ventricular outflow tract (RVOT) and left ventricular outflow tract (LVOT). The optimal R‐wave threshold was identified, compared with the V_2_S/V_3_R index, transitional zone (TZ) index, and V_2_ transition ratio, and validated in a prospective cohort study.

**Results:**

Lead I R‐wave amplitude for LVOT origins was significantly higher than that for RVOT origins (0.55 ± 0.13 vs. 0.32 ± 0.15 mV; *p* < .001). The area under the curve (AUC) for lead I R‐wave amplitude as assessed by receiver operating characteristic (ROC) analysis was 0.926, with a cutoff value of ≥0.45 predicting LVOT origin with 92.9% sensitivity and 88.2% specificity, superior to the V_2_S/V_3_R index, TZ index, and V_2_ transition ratio. VAs in the LVOT group mainly originated from the right coronary cusp (RCC) and left and right coronary cusp junction (L‐RCC). In the prospective study, lead I R‐wave amplitude identified the LVOT origin with 92.3% accuracy.

**Conclusion:**

Lead I R‐wave amplitude provides a useful and simple criterion to identify RCC or L‐RCC origin in OT‐VAs with lead V_3_ transition.

## 1 INTRODUCTION

Outflow tract ventricular arrhythmias (OT‐VAs) are the most common subgroup of idiopathic ventricular arrhythmias (VAs), with a high cure rate achieved by radiofrequency (RF) catheter ablation.^1^, ^2^, ^3^, ^4^ The majority (approximately 70%) of idiopathic VAs originate from the right ventricular outflow tract (RVOT).^5^ Less commonly, 10%–15% of cases arise from the left ventricular outflow tract (LVOT) and surrounding regions.^6^

VAs originating from the RVOT generally manifest a left bundle branch block (LBBB) electrocardiography (ECG) with R/S‐wave precordial transition at or after lead V_3_, while those originating from the LVOT manifest an earlier transition at or before lead V_3_.^7^ Although several criteria for ECG precordial leads have been proposed to differentiate the left from right origin, localizing the origin site of VAs with lead V_3_ transition can still be challenging.^8^, ^9^, ^10^, ^11^, ^12^, ^13^, ^14^

Lead I is a left‐sided lead, and the LVOT lies posterior and rightward relative to the RVOT.^15^ A negative complex in lead I typically suggests a site of origin (SOO) in the anterior RVOT, left coronary cusp (LCC), or adjacent left ventricle (LV) summit.^16^, ^17^ As the structure shifts more rightward, the QRS complex in lead I appears more positive. VAs originating from the LCC and LV summit commonly transit at lead V_1_ or V_2_.^18^ Thus, VAs with lead V_3_ transition and a positive complex in lead I, which usually originate from the RVOT, right coronary cusp (RCC), and the left and right coronary cusp junction (L‐RCC), need focused attention. We hypothesized that the R‐wave amplitude in lead I would be useful for differentiating the SOO.^19^

## 2 METHODS

### 2.1 Patient selection

Data of 346 consecutive patients (from January 2017 to September 2019) who underwent catheter ablation of OT‐VAs at the Second Hospital of Hebei Medical University were retrospectively reviewed. After excluding patients with structural abnormalities or ischemic heart disease (n = 32) or arrhythmogenic right ventricular cardiomyopathy (n = 1), paced rhythm (n = 16), failed ablation (n = 31), incomplete records (n = 51), precordial transition (from R/S < 1 to R/S ≥ 1) at lead V_1_ or V_2_ (n = 62), or transitional lead later than V_3_ (n = 44), we obtained ECG measurements from 109 cases of OT‐VAs with lead V_3_ transition. VAs with an R/S < 1 morphology in lead I (n = 27) were not included for analysis. Antiarrhythmic drugs were discontinued at least five half‐lives before the ablation. All patients gave informed consent for the procedure. This study complied with the Declaration of Helsinki and was approved by the Institutional Review Board of the Second Hospital of Hebei Medical University.

### 2.2 Electrophysiological study

A standard ten‐pole diagnostic catheter was positioned in the coronary sinus and a standard quadripolar diagnostic catheter was placed in the right ventricle through the femoral vein. Mapping and ablation were performed with an 8‐Fr, 3.5 mm‐tip irrigated THERMOCOOL or SMARTTOUCH Catheter (Biosense‐Webster, Diamond Bar, California, USA) via the right femoral vein (for the RVOT) or the right femoral artery (for the LVOT).

### 2.3 Mapping and ablation

**FIGURE 1:**
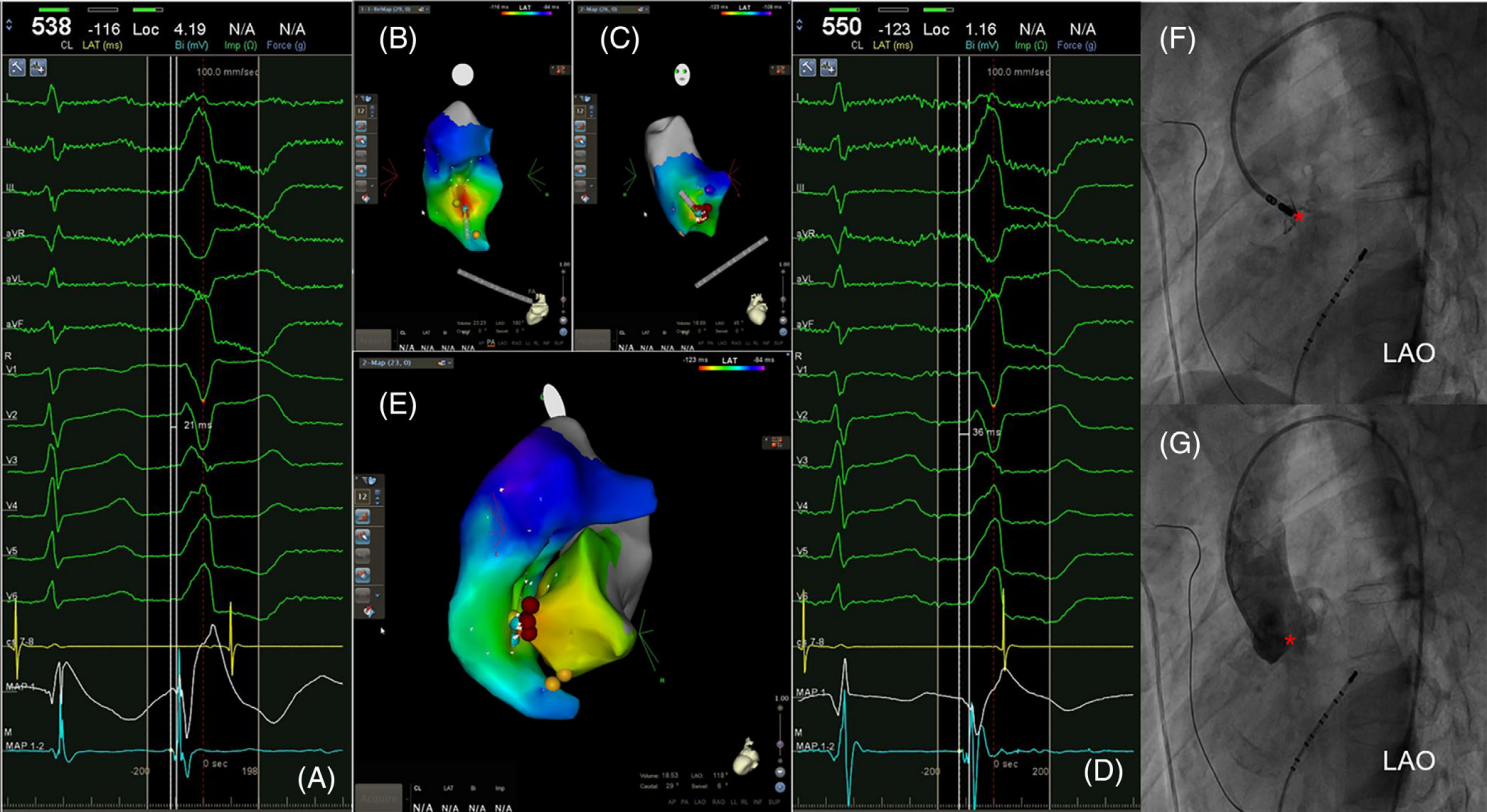
Example of mapping PVC origin and confirming successful ablation site. (A) and (B) The earliest activation site (EAS) in the septal RVOT preceding the QRS onset by 21 ms was not perfect. (C) and (D) Activation in LVOT preceding the QRS onset by 36 ms was earlier than in RVOT. (E) PVC was ablated successfully at L‐RCC. (F) Contrast agent injection through ablation catheter showed the catheter tip for the successful ablation site. (G) Angiography of aortic sinus with pigtail catheter. Red point: successful ablation target. Orange point: His bundle. Red star: successful ablation site. LAO, left anterior oblique. L‐RCC, left and right coronary cusp junction; LVOT, left ventricular outflow tract; PVC, premature ventricular contraction; RVOT, right ventricular outflow tract

The CARTO three‐dimensional (3D) electromagnetic mapping system (Biosense‐Webster) and standard fluoroscopy were used to localize the SOO of the VAs. An electroanatomic shell of the RVOT was created via the right femoral vein. Activation mapping was performed during frequently spontaneous premature ventricular contractions (PVCs) or ventricular tachycardia (VT). In cases with infrequent ectopy, isoproterenol was injected (1–20 μg/min) to promote triggered activity and manifest PVCs/VT. The target site for ablation was the earliest activation site (EAS), preceding QRS onset by at least 20 ms, along with a QS pattern in the unipolar electrogram. If the ablation in the RVOT was unsuccessful or no satisfactory RVOT sites were identified, we created the electroanatomic shell of the LVOT and mapped the LVOT sites via a retrograde aortic approach (Figure 1(A)‐(D)). If both LVOT and RVOT mapping failed, we mapped the great cardiac vein and/or the anterior interventricular vein. All mapping was performed after administering a heparin bolus to maintain an activated clotting time > 250 s. For sedation, Fentanyl was infused with a 1 μg/kg bolus followed by 1–2 μg/kg h administered intravenously.

The target site for catheter ablation was evaluated using the 3D electroanatomic mapping system (Figure 1(E)) with standard fluoroscopy. If the target site was localized at the aortic sinus, the 3D shell of each cusp was accurately reconstructed with the CARTO system. To confirm the catheter position within or between the cusps of the aortic sinus, we injected a contrast agent through the irrigated catheter followed by angiography of the aortic sinus with a pigtail catheter (Figure 1(F)‐(G)). In addition, selective angiography of the coronary artery or aorta was performed to assess the safe distance between the ablation site and these structures.

RF energy was delivered to the RVOT or LVOT using an irrigated 3.5 mm tip catheter with a target temperature of 43 °C and maximum power output of 30–35 W. The flow rate was 17–30 mL/min. RF energy delivery was continued for 40–60 s if an acceleration or reduction in the incidence of the PVCs/VT was observed within 10 s of starting ablation. Otherwise, RF delivery was terminated and the catheter was repositioned.

Successful catheter ablation was defined as: (1) absence of clinical VAs at 20 min after the last RF delivery during isoproterenol infusion; (2) absence of VAs in 24 h in‐hospital telemetry monitoring recordings after the procedure; (3) no recurrence of symptomatic arrhythmia as assessed by 24 h Holter 1 and 3 months after ablation. All patients with successful ablation were free of any antiarrhythmic drugs.

### 2.4 ECG measurement

Standard 12‐lead ECG electrode placement was used. The electrodes for the chest and limb leads were carefully placed by an experienced technician. The 12‐lead ECG morphologies of all patients during sinus rhythm (SR) and PVCs/VT were measured by two observers (who were blinded to the SOO) using electronic calipers on the recording system (the LEAD‐9000 Electrophysiology Management System, Jinjiang Electronic Science and Technology Co., Ltd, Sichuan, China). The lead gain was uniform with a paper speed of 100 mm/s. Amplitudes were measured using the vertical caliper tool and ratios were manually calculated. The mean value of two measurements was used for analysis. During both SR and PVCs/VT, the following measurements were obtained: (1) R‐ and S‐wave amplitudes in leads I, II, III, aVR, aVL, and V_1_ to V_3_; (2) R‐ and S‐wave duration in leads I, II, III, aVR, aVL, and V_1_ to V_3_; (3) Total QRS duration; (4) R amplitude ratio in leads III to II (III/II ratio), and Q amplitude ratio in leads aVL to aVR (aVL/aVR ratio); (5) V_2_S/V_3_R index, transitional zone (TZ) index, and V_2_ transition ratio. The T–P segment was considered the isoelectric baseline for measurement of R‐ and S‐wave amplitude. In cases with an RS pattern in lead I, R‐wave amplitude was defined as the difference between the highest and lowest point amplitude of the QRS complex. The total QRS duration was measured from the site of earliest initial deflection from the isoelectric line in any lead to the time of latest activation in any lead. The R‐wave duration was measured from the site of earliest initial deflection from the isoelectric line to the time at which the R‐wave intersected the isoelectric line. We standardized the SR measurements by measuring the largest R‐ and S‐waves over a 10‐s window at 25‐mm/s sweep speed to minimize respiratory variation. The V_2_S/V_3_R index was calculated by computing the S‐wave amplitude in lead V_2_ divided by the R‐wave amplitude in lead V_3_ during the VAs.^10^ The TZ index was calculated as follows: TZ score of the PVCs/VT minus the TZ score of the SR.^9^ The TZ score was graded with 0.5‐point increments according to the site of the R‐wave transition (e.g., TZ in V_2_ = 2‐point, V_2_‐V_3_ = 2.5‐point, V_3_ = 3‐point, and V_3_‐V_4_ = 3.5‐point). The V_2_ transition ratio was calculated as the percentage R‐wave during VT (R/R + S)_VT_ divided by the percentage R‐wave in SR (R/R + S)_SR_.^14^

### 2.5 Prospective analysis

To evaluate the reliability of lead I R‐wave amplitude, the measurement was repeatedly conducted in a prospective cohort of 39 patients with the same inclusion criteria between October 2019 and April 2020.

### 2.6 Statistical analysis

Continuous variables are expressed as mean ± 1 SD. Normally distributed continuous variables were compared using the Student *t* test, and nonnormally distributed variables were compared using the Mann–Whitney *U* test. Categorical variables were compared using the chi‐square test or Fisher's exact test. We performed receiver operating characteristic (ROC) curve analysis for prediction of the SOO to calculate the optimal threshold value for lead I R‐wave amplitude, as well as to compare the differences in accuracy among the alternative indices. The SPSS 24.0 software (SPSS Inc., Chicago, IL) was used for statistical analysis. A two‐tailed *p* value <.05 was considered significant.

## 3 RESULTS

### 3.1 Patient baseline and ECG characteristics

The baseline patient characteristics and electrocardiographic characteristics are given in Table 1. Between the RVOT and LVOT groups, there were no significant differences in gender, body mass index (BMI), left ventricular ejection fraction (LVEF), PVC burden, or VT classification. Lead I R‐wave amplitude was significantly higher in the LVOT group (0.55 ± 0.13 mV) compared with the RVOT group (0.32 ± 0.15 mV; *p* < .001). The R amplitude ratio in leads III to II was larger in the RVOT group compared with the LVOT group (0.91 ± 0.13 vs. 0.74 ± 0.18; *p* < .001). The Q amplitude in lead aVL was larger in the RVOT group (0.73 ± 0.23 mV) than in the LVOT group (0.51 ± 0.15 mV; *p* < .05). The Q amplitude ratio in leads aVL to aVR was also greater in the RVOT group (0.79 ± 0.22) than in the LVOT group (0.51 ± 0.20; *p* < .001). The V_2_S/V_3_R index was found to be lower in the LVOT group (1.47 ± 0.65) than in the RVOT group (2.13 ± 0.95; *p* < .05). Other parameters including the TZ index and V_2_ transition ratio did not show significant differences between these two groups.

**TABLE 1:** Baseline patient characteristics and electrocardiographic measurements

	Total (n = 82)	RVOT (n = 68)	LVOT (n = 14)	*p* value	AUC
Age (yrs)	51 ± 13	49 ± 13	57 ± 11	.020	
Male	25 (30.5%)	18 (26.5%)	7 (50.0%)	.155	
BMI (kg/m^2^)	25.32 ± 3.03	25.48 ± 3.08	24.55 ± 2.74	.298	
LVEF (%)	63.03 ± 6.22	63.36 ± 6.15	61.38 ± 6.52	.054	
PVC burden (mean n/24‐h Holter)	24 814 ± 13 791	25 102 ± 14 146	23 418 ± 12 293	.680	
PVC burden (%/24‐h Holter)	23.05 ± 12.30	23.15 ± 12.48	22.57 ± 11.86	.873	
Clinical arrhythmia					
Frequent PVCs	76 (92.7%)	64 (94.1%)	12 (85.7%)	.271	
Nonsustained VT	13 (15.9%)	9 (13.2%)	4 (28.6%)	.304	
Sustained VT	0	0	0		
Electrocardiographic measurements					
Total QRS duration of OT‐VA (ms)	176.27 ± 26.33	175.27 ± 27.08	181.14 ± 22.57	.451	0.558
Lead I					
R amplitude of OT‐VA (mV)	0.36 ± 0.17	0.32 ± 0.15	0.55 ± 0.13	.000	0.926
Lead II and III					
R amplitude of OT‐VA in lead II (mV)	1.57 ± 0.36	1.56 ± 0.39	1.59 ± 0.16	.234	0.601
R amplitude of OT‐VA in lead III (mV)	1.38 ± 0.41	1.42 ± 0.42	1.19 ± 0.33	.193	0.611
R amplitude ratio in leads III to II	0.88 ± 0.15	0.91 ± 0.13	0.74 ± 0.18	.000	0.782
Lead aVR and aVL					
Q amplitude of OT‐VA in lead aVR (mV)	0.96 ± 0.24	0.94 ± 0.25	1.03 ± 0.17	.074	0.652
Q amplitude of OT‐VA in lead aVL (mV)	0.69 ± 0.23	0.73 ± 0.23	0.51 ± 0.15	.001	0.798
Q amplitude ratio in leads aVL to aVR	0.74 ± 0.24	0.79 ± 0.22	0.51 ± 0.20	.000	0.832
Lead V_1_					
R amplitude of OT‐VA (mV)	0.29 ± 0.22	0.28 ± 0.15	0.33 ± 0.43	.686	0.542
S amplitude of OT‐VA (mV)	1.27 ± 0.46	1.26 ± 0.47	1.33 ± 0.44	.596	0.545
R duration of OT‐VA (ms)	54.51 ± 30.34	54.50 ± 24.67	54.56 ± 50.99	.997	0.522
Lead V_2_					
R amplitude of OT‐VA (mV)	0.57 ± 0.25	0.55 ± 0.24	0.66 ± 0.29	.115	0.628
S amplitude of OT‐VA (mV)	1.81 ± 0.81	1.87 ± 0.85	1.50 ± 0.55	.113	0.635
R duration of OT‐VA (ms)	62.29 ± 17.48	61.22 ± 18.21	67.49 ± 12.59	.224	0.631
R amplitude of SR (mV)	0.66 ± 0.33	0.63 ± 0.31	0.80 ± 0.43	.204	0.608
S amplitude of SR (mV)	1.10 ± 0.49	1.09 ± 0.51	1.11 ± 0.41	.936	0.507
Lead V_3_					
R amplitude of OT‐VA (mV)	0.98 ± 0.33	0.95 ± 0.30	1.11 ± 0.44	.091	0.608
S amplitude of OT‐VA (mV)	0.53 ± 0.33	0.55 ± 0.33	0.46 ± 0.34	.325	0.574
R duration of OT‐VA (ms)	102.98 ± 28.27	100.07 ± 28.54	117.12 ± 22.84	.028	0.687
Other parameters					
V_2_S/V_3_R	2.01 ± 0.93	2.13 ± 0.95	1.47 ± 0.65	.016	0.713
TZ index	0.10 ± 0.64	0.16 ± 0.65	−0.18 ± 0.50	.065	0.653
The V_2_ transition ratio	0.72 ± 0.37	0.71 ± 0.39	0.80 ± 0.30	.179	0.614

Abbreviations: AUC, area under the curve; BMI, body mass index; LVEF, left ventricular ejection fraction; LVOT, left ventricular outflow tract; OT‐VA, outflow tract ventricular arrhythmia; PVC, premature ventricular contraction; RVOT, right ventricular outflow tract; SR, sinus rhythm; TZ, transitional zone; VT, ventricular tachycardia.

### 3.2 Predicted accuracy of lead I R‐wave amplitude and other indices

**FIGURE 2:**
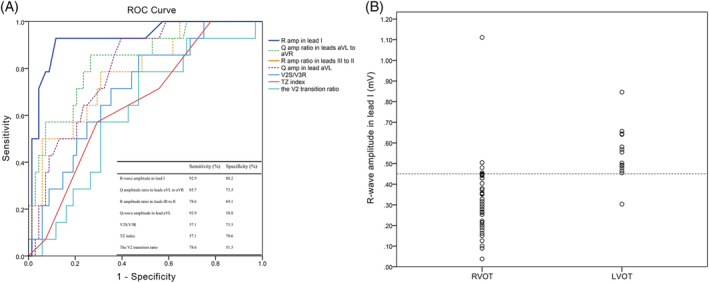
Predicted accuracy of R‐wave amplitude in lead I and other indices. (A) Receiver operating characteristic (ROC) analysis for lead I R‐wave amplitude and other parameters. The area under the curve (AUC) of lead I R‐wave amplitude (red line) was greatest with 0.926. (B) Scatterplot of lead I R‐wave amplitude in OT‐VAs originating from RVOT and LVOT. Dotted line indicates optimal cut‐off value of 0.45. LVOT, left ventricular outflow tract; OT‐VAs, outflow tract ventricular arrhythmia; RVOT, right ventricular outflow tract

The entire area under the curve (AUC) produced by ROC curve analysis is shown in Table 1. Among the ECG measurements and other indices, lead I R‐wave amplitude exhibited the largest AUC of 0.926 (Figure 2(A)). The optimal cut‐off value for predicting LVOT origin was 0.45, yielding 92.9% sensitivity and 88.2% specificity (Figure 2(B)). The AUC of the aVL/aVR ratio, III /II ratio and Q amplitude in aVL were 0.832, 0.782 and 0.798, and the cut‐off value was 0.66, 0.86, and 0.67, respectively. The accuracy of the R‐wave amplitude in lead I and other indices reported previously were calculated and are compared in Figure 2(A).

### 3.3 Successful ablation location and relationship with lead I R‐wave amplitude

Among 82 patients with transitional lead at V_3_ and a positive complex in lead I, 68 patients (82.9%) underwent successful ablation at the RVOT, and 14 patients (17.1%) at the LVOT. A total of 64 of 68 VAs originating from the RVOT were eliminated below or above the pulmonary valve. Of 14 cases originating at the LVOT, 3 (21.4%) were ablated in the RCC, 9 (64.3%) at the left and right coronary cusp junction (L‐RCC), and 2 (14.3%) in the LCC. Of 61 cases with lead I R‐wave amplitude <0.45 mV, the origins were as follows: 27 (44.3%) anteroseptal RVOT; 15 (24.6%) middle septal RVOT; 11 (18.0%) posterior septal RVOT; 6 (9.8%) free wall of RVOT; 1 (1.6%) pulmonary valve; and 1 LCC. Of 21 cases with lead I R‐wave amplitude ≥0.45 mV, 12 (57.1%) were successfully ablated from the RCC or L‐RCC, 7 (33.3%) were eliminated at the septum of the RVOT, 1 (4.8%) at the LCC, and 1 at the free wall of the RVOT.

### 3.4 Prospective validation cohort

The prospective cohort consisted of 39 consecutive idiopathic OT‐VA patients with lead V_3_ transition and a positive complex in lead I who underwent successful catheter ablation (12 male and 27 female). The mean age was 47 ± 13 years, and mean BMI was 23.62 ± 2.17 kg/m^2^. The mean PVC burden was 19.30 ± 8.12% with an ejection fraction of 60.95 ± 5.25%. 30 VAs were successfully ablated from the RVOT (anterior septum: 8; middle‐posterior septum:19; free wall: 3), and 9 from the LVOT (LCC: 2; RCC: 3; L‐RCC: 4). The R‐wave amplitude in lead I was able to correctly predict the SOO in 36/39 cases (92.3%).

## 4 DISCUSSION

### 4.1 Main findings

We present a simple and useful method of using lead I R‐amplitude to differentiate LVOT from RVOT origin for OT‐VAs with lead V_3_ transition. The R‐wave amplitude in lead I ≥ 0.45 mV suggests LVOT origin with 92.9% sensitivity, 88.2% specificity, and 92.3% accuracy in our validated cohort, which is more accurate than precordial lead indices such as the V_2_S/V_3_R and the TZ index, in addition to the V_2_ transition ratio. The measurement can be easily acquired with digital calipers on an electrophysiologic recording system. Because the measure is simple and does not require complex calculation, it might be especially helpful in clinical practice.

### 4.2 Anatomic considerations and R‐wave amplitude in lead I

**FIGURE 3:**
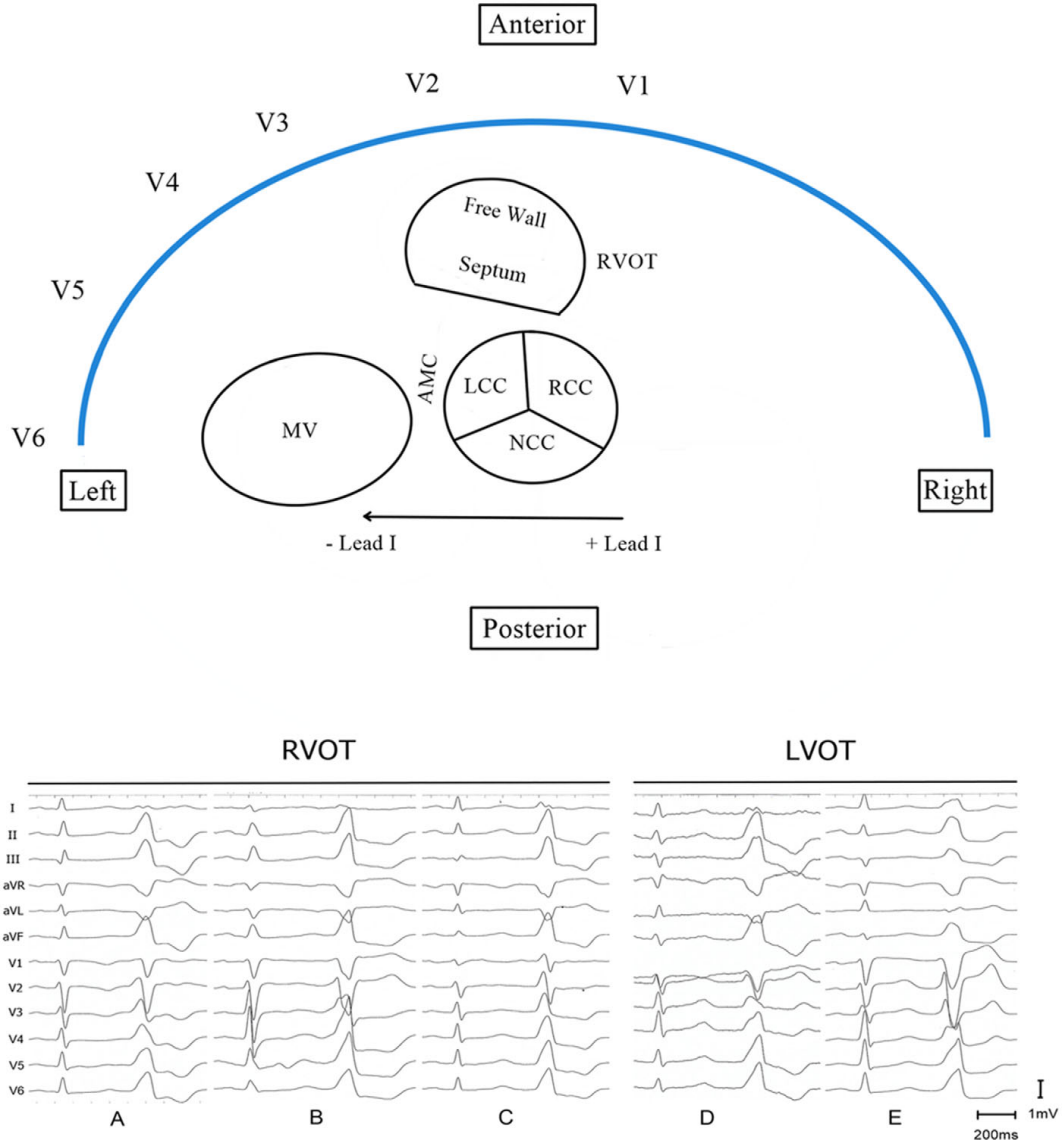
Schema indicating anatomical relationship between RVOT and LVOT; this affects ECG morphology of OT‐VAs with lead V_3_ transition. OT‐VAs were eliminated at anteroseptal RVOT (A), middle and posterior septal RVOT (B) and (C), LCC/RCC junction (D), and RCC (E). AMC, aortomitral continuity; LCC, left coronary cusp; LVOT, left ventricular outflow tract; MV, mitral valve; NCC, noncoronary cusp; OT‐VAs, outflow tract ventricular arrhythmias; RCC, right coronary cusp; RVOT, right ventricular outflow tract

The aortic root occupies the central location within the heart. The RVOT is located anterior to the LVOT, and passes leftward of the aortic root. When the aortic valve is viewed from the attitudinal orientation, the RCC is most anteriorly and rightward situated. The LCC lies posteriorly and leftward.^15^, ^18^ The middle‐posterior septal region of the RVOT comes in contact with the RCC and RCC/LCC commissure.^16^, ^20^ Lead I is a left‐sided lead and represents the horizontal dimension most conveniently. VAs originating from the left aspect of the outlets will appear flat or negative in lead I. As the origin site shifts more rightward, lead I appears progressively more positive (Figure 3). According to previous studies^21^, ^22^, ^23^, OT‐VAs with a QS pattern in lead I commonly originate from the supra‐pulmonic or anterior RVOT. For VAs with an R/S < 1 morphology in lead I, LCC, the aortomitral continuity (AMC) and summit can be common sites of origin, and usually present lead V_1_ or V_2_ transition. When lead I shows a RS (R/S > 1) or R pattern, VAs can be ablated from the middle‐posterior RVOT or aortic cusp, which requires extra measurements to differentiate left and right origin. Thus, we excluded cases with QS or R/S < 1 patterns in lead I and focused on positive lead I VAs. With the same tendency, VAs originating from the RCC and L‐RCC produce greater R‐wave amplitude in lead I than most originating from RVOTs in our study group, and we suggest that this is a useful index for differentiating the SOO.

### 4.3 Outlier and failed ablation cases

There were eight cases with lead I R‐wave amplitude ≥0.45 mV ablated from the RVOT for which we failed to localize the origin sites correctly with our criteria. In one of these cases, the VA was transiently eliminated from the RCC, with the site preceding QRS onset by 28 ms; the successful ablation site in the RVOT preceded QRS by 32 ms. The site of origin may have been located between the left and right outflow tracts. The BMI was higher or lower than the majority of individuals in 5/8 cases, which may result in anatomical variation. In 2/8 cases, the VAs were successfully ablated from the lower part of the posterior free wall of the RVOT; this SOO is connected to higher than usual R‐wave amplitudes in lead I.

A total of 13/31 failed cases exhibited lead V_3_ transition in precordial ECG. Three cases showed an R or Rs pattern in lead I. The R‐wave amplitudes in these three cases were 0.43, 0.54, and 0.55 mV, and the EASs were marked at the posterior septal RVOT, the posterior septal RVOT, and the L‐RCC, preceding the QRS onset by 35, 30, and 40 ms, respectively. The origin sites may have been located between the RVOT and LVOT, resulting in incomplete elimination of the VAs.

### 4.4 Comparison to other indices

Xie et al^17^ also evaluated lead I for differentiating left and right origin in OT‐VAs with left bundle branch block morphology and a right inferior axis (LBRI) pattern. They included cases with a QS pattern in lead I and recorded their R‐wave amplitude as 0mv, which accounted for 42/75 participants (56%) in the study. Thus, they measured smaller R‐wave amplitude in both the RVOT and LVOT groups compared with our study. The V_2_S/V_3_R index^10^ and TZ index^9^ were explored in OT‐VA patients with a LBRI pattern. In patients with lead V_3_ transition, the similar ECG morphologies in precordial leads may account for unideal values for differentiation. Betensky et al.^14^ reported that the V_2_ transition ratio predicted an LVOT origin with 91% accuracy in a 40‐member retrospective cohort and 21‐member prospective cohort with various morphologies in lead I. The population was limited and different, meaning that conclusions based on our cohort may be of limited accuracy because they could not be compared directly with previous studies.

In this study, the Q‐wave amplitude in lead aVL and ratio of leads aVL to aVR are also significantly larger in the RVOT than in the LVOT. Lead aVR/III and aVL/II have rightward and leftward vectors as additional horizontal approximations, respectively.^18^ Although lead I reflects the horizontal dimension better than leads aVL/II or aVR/III, corroborating lead I R‐wave amplitude with these indices to localize the SOO may also help to increase the accuracy.

## 5 LIMITATIONS

In this study, several insufficiencies should be taken into consideration before attempting to apply the results. Cases with structural heart disease that affect cardiac anatomy were excluded. Other factors like BMI that have a potential influence on the attitudinal orientation of the heart did not produce significant differences between the LVOT and RVOT groups in this retrospective study. However, we did not obtain cross‐sectional cardiac images to exclude inter‐individual variables. This may explain a few cases that did not match our criteria. Additionally, we used the location of the successful catheter ablation as the VA's site of origin. It is possible that the ablation lesions extended into adjacent structures and that VAs from the LVOT could be abolished via the RVOT. Finally, due to the unreliability of pace mapping for VAs originating from the aortic sinus,^24^ our study may be not suitable for pace mapping cases.

## 6 CONCLUSION

Lead I R‐wave amplitude provides a simple and useful criterion to distinguish LVOT from RVOT origin in VAs with lead V_3_ transition and a positive complex in lead I. OT‐VAs with lead V_3_ transition and greater R‐wave amplitude (≥0.45 mV) may originate from the RCC or L‐RCC. VAs with lead V_3_ transition and smaller R‐wave amplitude suggest an origin site in the RVOT.

## CONFLICT OF INTEREST

The authors declare no potential conflict of interest.

## Data Availability

The datasets generated and/or analyzed during the current study are available from the corresponding author, Suyun Liu, upon reasonable request.
